# Stereotactic radiosurgery combined with targeted/ immunotherapy in patients with melanoma brain metastasis

**DOI:** 10.1186/s13014-020-1485-8

**Published:** 2020-02-14

**Authors:** Indrawati Hadi, Olarn Roengvoraphoj, Raphael Bodensohn, Jan Hofmaier, Maximilian Niyazi, Claus Belka, Silke Birgit Nachbichler

**Affiliations:** 1 Department of Radiation Oncology, University Hospital, LMU Munich, Munich, Germany; 2 German Cancer Consortium DKTK, Munich, Germany

**Keywords:** Brain metastasis, Immunotherapy, Melanoma, Stereotactic radiosurgery

## Abstract

**Background:**

There is limited data on the use of targeted or immunotherapy (TT/IT) in combination with single fraction stereotactic radiosurgery (SRS) in patients with melanoma brain metastasis (MBM). Therefore, we analyzed the outcome and toxicity of SRS alone compared to SRS in combination with TT/IT.

**Methods:**

Patients with MBM treated with single session SRS at our department between 2014 and 2017 with a minimum follow-up of 3 months after first SRS were included. The primary endpoint of this study was local control (LC). Secondary endpoints were distant intracranial control, radiation necrosis-free survival (RNFS), and overall survival (OS). The local/ distant intracranial control rates, RNFS and OS were analyzed using the Kaplan-Meier method. The log-rank test was used to test differences between groups. Cox proportional hazard model was performed for univariate continuous variables and multivariate analyses.

**Results:**

Twenty-eight patients (17 male and 11 female) with 52 SRS-lesions were included. The median follow-up was 19 months (range 14–24 months) after first SRS. Thirty-six lesions (69.2%) were treated with TT/IT simultaneously (4 weeks before and 4 weeks after SRS), while 16 lesions (30.8%) were treated with SRS alone or with sequential TT/IT. The 1-year local control rate was 100 and 83.3% for SRS with TT/IT and SRS alone (*p* = 0.023), respectively. The estimated 1-year RNFS was 90.0 and 82.1% for SRS in combination with TT/IT and SRS alone (*p* = 0.935). The distant intracranial control rate after 1 year was 47.7 and 50% for SRS in combination with TT/IT and SRS alone (*p* = 0.933). On univariate analysis, the diagnosis-specific Graded Prognostic Assessment including the BRAF status (Melanoma-molGPA) was associated with a significantly improved LC. Neither gender nor SRS-PTV margin had a prognostic impact on LC. V10 and V12 were significantly associated with RNFS (*p* < 0.001 and *p* = 0.004).

**Conclusion:**

SRS with simultaneous TT/IT significantly improved LC with no significant difference in radiation necrosis rate. The therapy combination appears to be effective and safe. However, prospective studies on SRS with simultaneous TT/IT are necessary and ongoing.

**Trial registration:**

The institutional review board approved this analysis on 10th of February 2015 and all patients signed informed consent (UE nr. 128–14).

## Background

Among all malignancies, melanoma has the highest risk to spread to the brain besides lung cancer [1]. Thirty-four percent of patients with melanoma developed brain metastases in clinical studies and 75% of melanoma patients were found to have brain metastases in autopsy studies [2]. With a median overall survival of 4.6 months brain metastases are the leading cause of death in melanoma patients [3]. Because of its resistance to radio- and chemotherapy, the management of melanoma brain metastases remains challenging. Standard local treatment options for MBM are stereotactic radiosurgery (SRS), resection, or whole brain radiotherapy (WBRT). In the past, WBRT has been the standard therapy for multiple brain metastases. Nevertheless, SRS is nowadays preferred over WBRT, especially because of preserving neurocognition and because of its excellent local control rates [4]. SRS might also have synergistic effects with systemic therapy [5]. There are certain cases, that SRS could not be performed, for example due to numerous (> 10 lesions) and large brain metastases. In this situation, WBRT with hippocampal sparing combined with simultaneous integrated boost of the metastases enables better outcomes with less neurological toxicity [6, 7].

The use of targeted therapies and immune check point inhibitors, such as BRAF inhibitors, MEK inhibitors, CTLA-4 antibodies and PD-1 antibodies has changed the paradigm in metastatic melanoma patients in recent years. These systemic therapies showed to improve overall survival of patients with metastatic melanoma significantly [8].

SRS combined with immuno- or targeted therapy might be more effective than SRS alone [9]. Still, there is limited data on the effects of targeted/ immunotherapy (TT/IT) in combination with SRS as only few studies evaluated TT/IT in MBM and several studies for TT/IT excluded patients with MBM [10, 11]. This study aims to analyze the outcome and toxicity of SRS alone compared with SRS and concurrent TT/IT.

## Patients and methods

### Patients

Patients with MBM who had been treated with single session SRS at our department between 2014 and 2017 were retrospectively identified from the institutional database. Only patients with a minimum follow-up of 3 months were included in the analysis. Patient, tumor, and treatment information were extracted from the charts. Treatment groups were categorized as SRS alone, and SRS plus concurrent TT/IT. Furthermore, the patients were assessed using the diagnosis-specific prognostic index for patients with MBM. The previous Melanoma-Graded Prognostic Assessment (Melanoma-GPA) included age, Karnofsky Performance Score (KPS), the absence or presence of extracranial metastases and the number of brain metastases. BRAF status had been found to be of prognostic significance in the latest update and therefore had been incorporated as an additional factor into the new Melanoma-molGPA [12].

The institutional review board approved this analysis and all patients signed informed consent prior to the start of therapy (UE nr. 128–14).

### Targeted/ immunotherapy (TT/IT)

TT/IT was considered as concurrent if it was applied within 4 weeks before or after SRS. BRAF inhibitors (dabrafenib, vemurafenib, encorafenib), MEK inhibitors (trametinib, binimetinib), CTLA-4 antibody ipilimumab, IgG4 anti-PD-1 antibody nivolumab and PD-1 antibody pembrolizumab were registered as TT/IT.

### SRS technique

Single session SRS treatment planning included fusion of T1-weighted gadolinium enhanced magnetic resonance imaging (MRI) of the brain with 1 mm slice thickness with computed tomography (CT) simulation imaging. Patients have been immobilized in an invasive stereotactic head frame until 2014, afterwards a noninvasive thermoplastic mask system (double or Brainlab) has been used. From 2014 until 2015, a uniform 3 mm expansion of the GTV was used to create the PTV. This margin expansion was initially defined as our institutional standard taking into consideration positional deviation due to setup, mechanical and imaging errors during the irradiation period, and the assumed microscopic extension of the tumor around the GTV [13]. With the improvement of IGRT, GTV was expanded by 2 mm uniformly to create the PTV after 2015. Another margin reduction to 1 mm from GTV to PTV was implemented at our institution after 2017, which was not included in this analysis.

Prescription doses were determined by the treating physician based on the diameter of the metastases and the location of the lesion: metastases with a diameter of at least 4 mm and not exceeding 25 mm were treated with 18–20 Gy prescribed to the 80% isodose. Dose might be reduced because of previous irradiation, critical tumor location or irregular shape. Dose escalation to 24 Gy was allowed in uncritical small lesions. Hypofractionated stereotactic radiotherapy (SRT) was performed for metastases with diameter greater than 30 mm, which was not included in this analysis. Radiation therapy was delivered using a linear accelerator (LINAC) with a maximum energy of 6 MV. After 2016, plans were delivered in flattening-filter-free (FFF) mode and with a maximum energy of 10 MV. Image guidance has been provided with cone beam CT and additionally with the Brainlab ExacTrac positioning system since November 2014.

### Follow-up

MRI at 2–3 months intervals as well as neurological status assessment were done to follow-up the patients after treatment. According to RECIST criteria, local failure was defined as a ≥ 20% radiographic increase in the size of the previously irradiated area, which remained consistent or continued to increase on subsequent imaging [14]. Radiation necrosis was defined as a lesion with certain radiographic changes in MRI. These changes might be obvious at least 3 month post-SRS and were described as a peripheral enhancing and central necrotic lesion on T1-weighted post-gadolinium MRI sequences, as well as an increase in volume of the lesion within the high dose SRS area [15]. Subsequent metabolic imaging with 18F-Fluoro-Ethyl-Tyrosine positron-emission tomography (FET-PET) and stereotactic biopsy were performed to confirm the diagnosis of brain necrosis. The case of a patient with suspected RN after SRS has been discussed in the multidisciplinary tumor board with a neuroradiologist, a radiation oncologist, and a neurosurgeon to meet a consensus regarding diagnosis and treatment. RN was considered symptomatic if patients presented with neurologic symptoms, such as headache, nausea, vertigo, or other neurological deficits. Steroids or bevacizumab in case of steroid-refractory RN were used to treat symptomatic RN [16].

### Statistical analysis

For patients with SRS for multiple lesions each lesion was analyzed independently.

The primary endpoint of this study was local BM control, which included all treated lesions not meeting the definition of local failure.

Secondary endpoints were distant intracranial control (DIC), radiation necrosis-free survival (RNFS), and overall survival (OS). DIC was defined as freedom from development of new BM or leptomeningeal disease outside of the irradiated volume. The local control rates were considered as the interval between the date of SRS and the date of local failure, or date of last follow up. The distant intracranial control rates were defined as the interval between the date of the first SRS and the date of brain MRI with development of new brain metastasis, or date of last follow-up. RNFS was defined as the interval between the date of SRS and the date of diagnosis of radiation necrosis, or date of last follow-up. OS was considered as the interval between the date of MRI with first diagnosis of brain metastasis and the date of death of any cause, or date of last follow-up. These endpoints were analyzed using the Kaplan-Meier method. The log-rank test was used to test differences between groups. Patient demographics were calculated using descriptive statistics as absolute and relative frequencies. Cox proportional hazard model was performed for univariate continuous variables. A two tailed *p*-value of < 0.05 was considered significant. Statistical analyses were done with IBM SPSS Statistics, Version 25 (IBM, Armonk, New York, USA).

## Results

### Patient characteristics

Thirty patients with 55 melanoma brain metastases were treated with SRS at our institution between 2014 and 2017. Patients with a follow-up shorter than 3 months were excluded, resulting in 28 patients and 52 lesions for analyses. Patients had a median age of 61 years (range 19–86 years) and developed brain metastasis with a median of 25.5 months after first diagnosis of melanoma (range 0–137 months).

Twenty-two patients (78.6%) had a KPS of 90–100%, 5 patients (17.9%) of 80%, and one patient (3.6%) of 70%. Extracranial systemic disease was controlled in 10 patients (35.7%). BRAF-mutation (mostly V600E) was found in 14 patients (50.0%).

Regarding TT/IT, two patients (7.1%) were treated with BRAF inhibitors (dabrafenib or vemurafenib), one patient was treated with trametinib (3.6%), 2 patients received ipilimumab (7.1%), nivolumab or pembrolizumab was given in 6 patients (21.4%). The combination of BRAFi and MEKi (dabrafenib-trametinib or encorafenib-binimetinib) was given in 3 patients (10.7%). Nivolumab-ipilimumab was given in 7 patients (25.0%). One patient (3.6%) received a BRAFi and MEKi combination at the beginning, the therapy regimen was then changed to anti-PD-1 due to progressive disease. Another patient (3.6%) received also a BRAFi and MEKi combination at the beginning, which was then changed to a triple combination (pembrolizumab, dabrafenib, and trametinib) due to progression. Thus, 20 patients (71.4%) were treated with SRS and concurrent TT/IT.

Five patients (17.9%) had no TT/IT at all, because brain metastases were diagnosed in recurrence-free and treatment-free interval. TT/IT was not applied concurrently in 3 patients.

Patients’ characteristics are summarized in Table 1.

**Table 1:** Patients` characteristics

Characteristic	Number of patients absolute relative	
Sex		
Female	11	39.3%
Male	17	60.7%
Karnofsky performance status (KPS)		
90–100%	22	78.6%
80%	5	17.9%
70%	1	3.6%
BRAF Status		
BRAF V600-E-Mutation	14	50.0%
No BRAF V600-E-Mutation	14	50.0%
Controlled extracranial disease		
Yes	10	35.7%
No	18	64.3%
RPA class		
1	6	21.4%
2	22	78.6%
3	0	0%
Melanoma-molGPA		
0.5–1.0	2	7.1%
1.5–2.0	11	39.3%
2.5–3.0	11	39.3%
3.5–4.0	4	14.3%
Number of SRS lesions per patient		
1	13	46.4%
2	7	25.0%
3	7	25.0%
4	1	3.6%
Whole brain radiotherapy		
Yes	4	14.3%
*Before first SRS*	*1*	*3.6%*
*After first SRS*	*3*	*10.7%*
No	24	85.7%
Targeted/ immunotherapy (TT/IT)		
BRAFi *(Dabrafenib/ Vemurafenib)* MEKi *(Trametinib)*	2 1	7.1% 3.6%
Anti-CTLA-4 *(Ipilimumab)*	2	7.1%
Anti-PD1 *(Nivolumab/Pembrolizumab)*	6	21.4%
BRAFi+MEKi *(Dabrafenib-Trametinib/ Encorafenib-Binimetinib)*	3	10.7%
BRAFi+MEKi ➔ Anti-PD1	1	3.6%
Anti-PD1 + Anti-CTLA-4 *(Nivolumab-Ipilimumab)*	7	25.0%
BRAFi+MEKi ➔Triple combination (*Pembrolizumab-Dabrafenib-Trametinib)*	1	3.6%
none	5	17.9%
Concurrent TT/IT		
Yes	20	71.4%
No	8	28.6%

### Lesion characteristics and treatment parameters

Fifty-two lesions with a median follow-up of 19 months (range 14–24 months) after SRS were analyzed. Median lesion diameter was 8 mm (range 4–23 mm), median GTV volume 0.4 ccm (range 0.08–6.54 ccm), and median PTV volume 1.6 ccm (range 0.30–13.46 ccm). The most common SRS doses were 20 Gy (*n* = 30; 57.7%) and 18 Gy (*n* = 17; 32.7%) prescribed at the 80% isodose line (*n* = 51, 98.1%). One lesion was treated with SRS dose reduction to 15Gy because of previous WBRT with boost adjacent to the current lesion.

Regarding the fixation technique, an invasive stereotactic head frame was used in 5 lesions (9.6%), a frameless Brainlab mask in 9 (17.3%), and a frameless double thermoplastic mask in 38 (73.1%). Treatment planning was done with Oncentra MasterPlan® in 30 lesions (57.7%), Monaco® in 17 (32.7%), and Radionics Xplan in 5 (9.6%).

Five lesions in 2 patients were treated with WBRT before SRS and 3 lesions in 3 patients received WBRT afterwards.

SRS only was performed in 10 lesions (19.2%). TT/IT was applied before SRS in 24 lesions (46.2%), the median duration from application of TT/IT to SRS was 9.5 days (range 3–43 days). TT/IT was given after SRS in 39 lesions (75.0%) with a median duration of 9 days from SRS to application of TT/IT (2–97 days).

We defined concurrent TT/IT as a TT/IT, which was given within 4 weeks before and/or after SRS. According to this definition, TT/IT was given concurrently before SRS in 21 lesions (40.4%, median: 8 days, range 3–21 days). Additionally, further 15 lesions received TT/IT after SRS, resulting in 36 lesions (69.2%), which were treated with TT/IT concurrently (median: 8 days, range 2–27 days, before and/or after SRS). Lesion characteristics and treatment parameters are summarized in Table 2.

**Table 2:** Lesion characteristics and treatment parameters

Parameter	Number of lesions absolute relative	
Number of lesions with follow up ≥3 months	52	100%
Location of metastasis		
Left	26	50.0%
Right	25	48.1%
Midline	1	1.9%
Frontal	19	36.5%
Parietal	6	11.5%
Temporal	8	15.4%
Occipital	6	11.5%
Frontotemporal	1	1.9%
Temporoparietal	1	1.9%
Parietooccipital	1	1.9%
Cerebellum	3	5.8%
Others (basal ganglia, insular cortex, parahippocampal, parafalcin)	7	13.5%
GTV to PTV margin (mm)		
2 mm	24	46.2%
3 mm	28	53.8%
Prescribed dose (Gy)		
15	1	1.9%
17	2	3.8%
18	17	32.7%
20	30	57.7%
24	2	3.8%
Prescribed isodose (%)		
70	1	1.9%
80	51	98.1%
Treatment planning software		
Oncentra Masterplan	30	57.7%
Monaco	17	32.7%
Radionics	5	9.6%
Fixation technique		
Frameless Brainlab mask	9	17.3%
Frameless double thermoplastic mask	38	73.1%
Invasive fixed head frame	5	9.6%
Targeted/ immunotherapy (TT/IT)		
BRAFi	5	9.6%
MEKi	1	1.9%
Anti-CTLA-4	4	7.7%
Anti-PD1	14	26.9%
BRAFi+MEKi	6	11.5%
Anti-PD1 + Anti-CTLA-4	10	19.2%
Triple combination	2	3.8%
none	10	19.2%
Application of TT/IT		
Before SRS (median: 9.5 days, range: 3-43 days)	24	46.2%
Concurrent before SRS (median: 8 days, range: 3–21 days)	21	40.4%
After SRS (median: 9 days, range: 2–97 days)	39	75.0%
Concurrent after SRS (median: 8 days, range: 2–27 days)	15	28.8%
Concurrent (4 weeks before or 4 weeks after SRS)	36	69.2%

### Local control

**Fig. 1:**
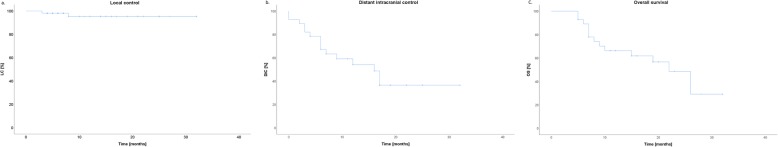
Kaplan-Meier plots of local control (LC), distant intracranial control (DIC) and overall survival (OS) for all patients with melanoma brain metastases treated with stereotactic radiosurgery (SRS). One- and 2-year LC rates after SRS were 98.1 and 95.4%, respectively **(a)**. One- and 2-year DIC rates after SRS were 54.2 and 36.6%, respectively, median 16 months **(b)**. Median overall survival was 22 months after first diagnosis of brain metastasis. One– and 2-year OS rates were 66.3 and 48.6% after first diagnosis of brain metastasis **(c)**

Two lesions developed local failure after SRS (3.8%). Local control rates at 1 and 2 years for all patients with MBM treated with SRS were 98.1 and 95.4%, respectively. Median local control has not been reached after a median follow-up of 14 months (range: 3–32 months) **(**Fig. 1a**).**

**Fig. 2:**
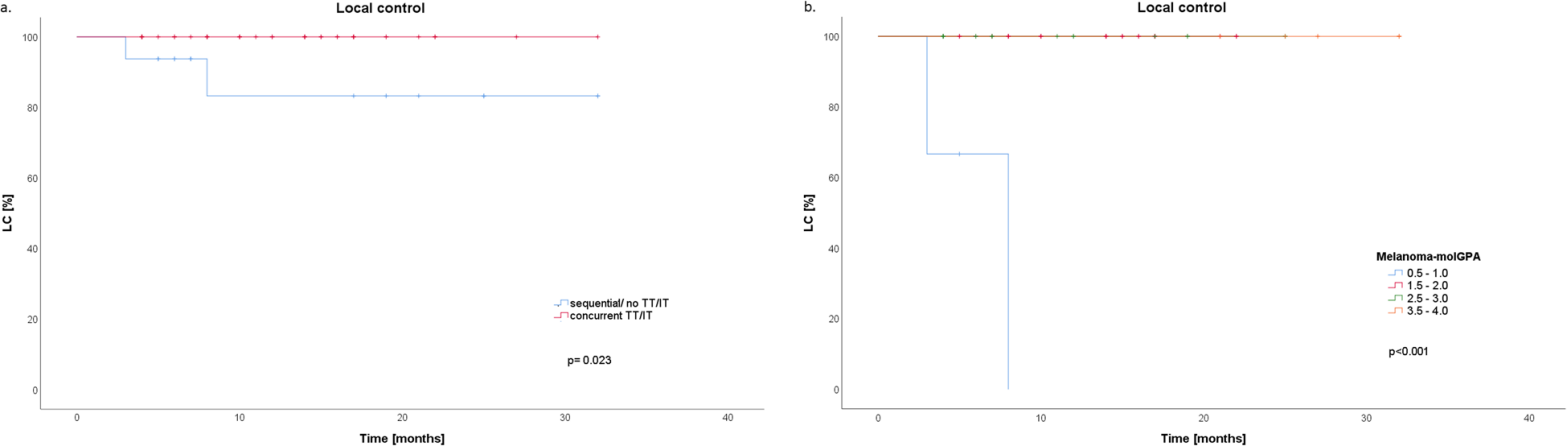
On univariate analysis, the 1-year local control (LC) rates for stereotactic radiosurgery (SRS) with concurrent targeted/ immunotherapy (TT/IT) and SRS alone were 100 and 83.3% (*p* = 0.023) **(a)**. Melanoma-molGPA as another prognostic factor was also associated with improved LC (*p* < 0.001) **(b)**

One-year LC rates were 100 and 83.3% for SRS with concurrent TT/IT and SRS alone (*p* = 0.023) **(**Fig. 2a**)**. Furthermore, the timing of concurrent TT/IT was analyzed; it showed that, TT/IT which was given *after* SRS was associated with improved 1-year LC (100 and 83.3% for concurrent TT/IT *after* SRS and SRS alone, *p* < 0.023). Concurrent TT/IT given *before* SRS did not gain prognostic significance for 1-year LC (91.4 and 100% for concurrent TT/IT *before* SRS and SRS alone, *p* = 0.197). On further univariate analysis, Melanoma-molGPA was associated with significantly improved LC **(**Fig. 2b**).** Gender, BRAF status, PTV margin, V10 and V12 did not gain prognostic significance for LC (Table 3**)**.

**Table 3:** Univariate analyses of local control (LC) and radiation necrosis-free survival (RNFS) of the 52 lesions

Variable	Lesions (n)	1 yr-LC (%)	*p*-value	1 yr-RNFS (%)	*p*-value
Gender					
Male	33	95.7		84.3	
Female	19	94.7	0.688	94.4	0.165
PTV margin					
2 mm	24	100		86.3	
3 mm	28	90.8	0.688	90.0	0.407
Targeted/immunotherapy (TT/IT)					
Concurrent	36	100		90.0	
No/ sequential	16	83.3	*0.023*	82.1	0.935
Concurrent TT/IT before SRS					
Yes	21	91.4		78.3	
No	31	100	0.197	86.9	0.723
Concurrent TT/IT after SRS					
Yes	36	100		90.0	
No	16	83.3	*0.023*	82.1	0.935
BRAF					
BRAF wild type	27	91.5		91.6	
BRAF mutation	25	100	0.189	81.9	0.151
Melanoma-molGPA					
0.5–1.0	3	0		50.0	
1.5–2.0	27	100		90.9	
2.5–3.0	16	100		91.7	
3.5–4.0	6	100	*< 0.001*	83.3	0.147
V10					
**≤** 12 ccm	40	94.6		94.4	
**>** 12 ccm	12	100	0.55	37.0	*< 0.001*
V12					
**≤** 10 ccm	41	94.8		91.7	
**>** 10 ccm	11	100	0.587	70.0	*0.004*

### Distant intracranial control and overall survival

Distant intracranial failure was found in 15 of the 28 patients (53.6%). Median time to distant intracranial failure was 16 months after first SRS treatment. One- and 2-year DIC rates were 54.2 and 36.6%, respectively **(**Fig. 1b**)**. DIC rates after 1 year were 47.7% und 50% for SRS with concurrent TT/IT and SRS alone (*p* = 0.933). Due to new intracranial metastases, WBRT was applied in 3 patients and 12 patients underwent additional SRS for new metastases. A higher number of brain metastases at first diagnosis (> 3 metastases) was associated with significantly worse distant intracranial control (*p* = 0.011) in univariate analysis. Gender, BRAF status, Melanoma-molGPA, and concurrent TT/IT were not of prognostic significance for distant intracranial control **(**Table 4**)**.

**Table 4:** Univariate analyses of distant intracranial control (DIC) and overall survival (OS) of the 28 patients

Variable	Patients (*n*=)	1 yr-DIC (%)	*p*-value	1 yr-OS (%)	*p*-value
Gender					
Male	17	49.2		61.2	
Female	11	60.6	0.216	72.7	0.426
BRAF status					
BRAF V600-E-Mutation	14	40.1		63.5	
No BRAF V600-E-Mutation	14	54.4	0.297	69.6	0.382
Melanoma-molGPA					
0.5–1.0	2	50.0		0	
1.5–2.0	11	32.7		14.5	
2.5–3.0	11	49.7		53.0	
3.5–4.0	4	50.0	0.535	75.0	0.087
Number of brain metastases					
1	12	47.6		72.7	
2	7	53.6		57.1	
3	6	62.5		50.0	
> 3	3	0	*0.011*	0	0.629
Targeted/ immunotherapy					
Concurrent	19	47.7		64.8	
No/ sequential	9	50.0	0.933	55.6	0.233

Median OS was 22 months after first diagnosis of brain metastasis. One– and two-year-OS rates were 66.3 and 48.6% after first diagnosis of brain metastasis **(**Fig. 1c**)**. Patients with better Melanoma-molGPA-score showed a tendency for better OS (*p* = 0.087). Other factors (gender, BRAF status, number of brain metastases, and concurrent TT/IT) did not gain prognostic significance for OS (Table 4**)**.

### Radiation necrosis (RN)

**Fig. 3:**
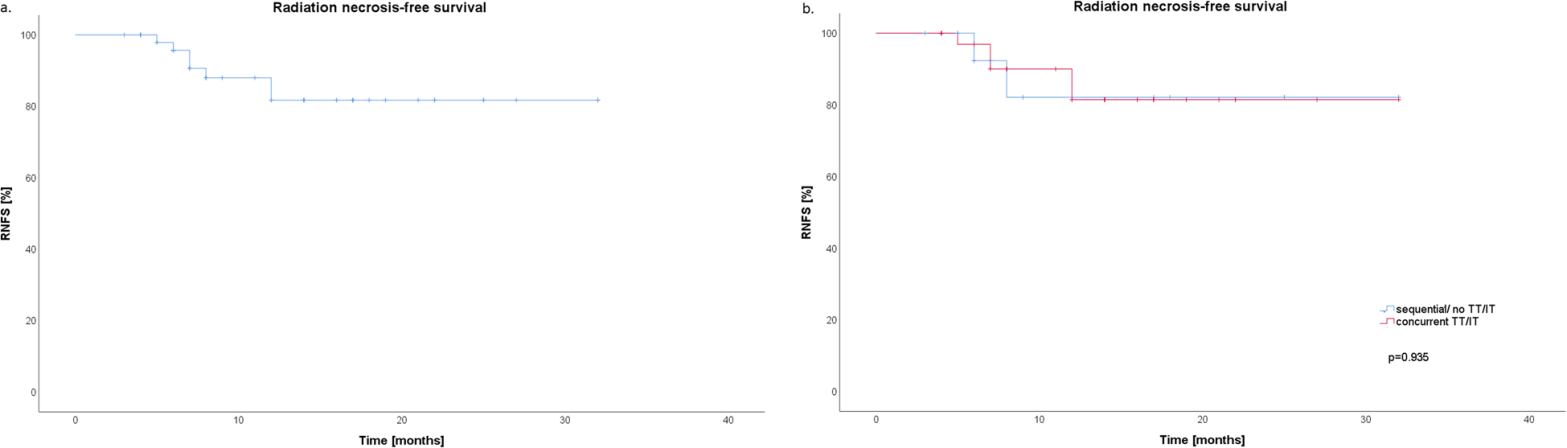
Radiation necrosis-free survival (RNFS) rates for all patients after 1 and 2 years were 87.9 and 81.7%, respectively **(a)**. The estimated 1-year RNFS rates were 90.0 and 82.1% for stereotactic radiosurgery (SRS) with concurrent targeted/ immunotherapy (TT/IT) and SRS alone (*p* = 0.935) **(b)**

Symptomatic radiation necrosis was found in 7 lesions (13.5%). All patients underwent metabolic FET-PET imaging and were treated with steroids or bevacizumab in case of steroid refractory symptoms or steroid induced side effects. Radiation necrosis-free survival (RNFS) rates after one and 2 years were 87.9 and 81.7%, respectively (Fig. 3a**)**. The estimated 1-year RNFS rates were 90.0 and 82.1% for SRS with concurrent TT/IT and SRS alone (*p* = 0.935) **(**Fig. 3b**)**. We analyzed concurrent TT/IT given before and after SRS, neither of these factors are significant for radiation necrosis development (*p* = 0.723 and p = 0.935). The timing of targeted therapy given before SRS was further analyzed from 7 days until 2 days before SRS, but was not prognostic for brain necrosis.

On further univariate analysis, the volume of normal brain tissue which received ≥10 Gy and ≥ 12 Gy (V10 and V12) was significantly associated with the occurrence of radiation necrosis (*p* < 0.001 and *p* = 0.004) (Table 3**)**.

## Discussion

In our analysis of SRS combined with TT/IT in MBM, we reported several findings: (i) LC was significantly improved in the cohort of SRS and concurrent TT/IT, (ii) distant intracranial control and OS were not significantly different in SRS with concurrent TT/IT and SRS alone, (iii) radiation necrosis rate was not significantly different in the two groups.

We reported an excellent 1-year local control rate of 98.1% and local failure has been found in only two lesions. TT/IT concurrent with SRS revealed a statistically significant better LC than non-concurrent TT/IT /SRS alone (one-year LC 100% versus 83%; *p* = 0.023). This finding is in accordance with preceding studies, which showed a statistically significant difference in LC favoring concurrent therapy [17–19]. Our analysis also confirmed that Melanoma-molGPA, which incorporated BRAF mutation status into its calculation, is a prognostic factor for LC. Similar results have been shown in previous studies [12, 20].

After the diagnosis of brain metastases, our cohort showed 1-year OS rates of 66.3% in both groups, which is in accordance with other analyses [17, 21–23]. A significant OS difference, with OS improvement in SRS and concurrent TT/IT has been described in other analyses [5, 17, 19, 20, 24]. Our analysis showed a one-year-OS rate of 68.2% in the group concurrent TT/IT and SRS and of 50% in the sequential TT/IT /SRS alone group. However, we could not find a significant difference in OS between these two groups, probably because of the small numbers of patients in our analysis.

We found 1- and 2-year distant intracranial control rates of 54.4 and 36.6%, which were comparable to and slightly better than other reports [25]. However, we were not able to show a significant difference in DIC between the sequential TT/IT /SRS alone group and the concurrent TT/IT group assumably because 4 patients (14.3%) received WBRT in addition to SRS and due to the limited number of patients included in our analysis.

Regarding the treatment sequencing, previous studies suggested that TT/IT should be administered within 4 weeks of SRS, as this was associated with better intracranial control and survival compared to SRS alone or non-concurrent TT/IT [5, 26]. In an analysis from Minniti et al., nivolumab or ipilimumab were administered with a median interval of 3 days from SRS [27]. This study showed that a concurrent SRS with nivolumab or ipilimumab had a meaningful intracranial activity, with an 1-year intracranial PFS rate of 42% with nivolumab and of 17% with ipilimumab. Minniti et al. also reported that, a subset of patients (15%) developed a symptomatic radiation necrosis. With a median follow up of 14 months, symptomatic RN was found in 7 patients (13.5%) in our cohort. This RN rate is comparable with the aforementioned study. There were no significant differences regarding RN rate between the SRS alone/sequential TT/IT group and the SRS with concurrent TT/IT group in our analysis. RNFS in patients treated with SRS with concurrent TT/IT was comparable to an historically reported rate of RNFS in SRS alone cohorts [28, 29]. Kohutek et al. reported an actuarial incidence of radiation necrosis of 17.2% at 12 months after SRS alone [28] and Minniti et al. reported that brain necrosis occurred in 24% of treated lesions after SRS alone [29]. Nevertheless, it is worth mentioning, that a wider PTV margin (2–3 mm) was applied in our SRS cohort. There was no statistical significant difference regarding brain necrosis between a 2 mm and a 3 mm margin in our analysis. However, Kirkpatrick et al. revealed that radiation necrosis was more frequently seen in the 3-mm arm instead of the 1-mm arm with a low rate of local recurrence in both arms [30]. Therefore, we adopted the 1-mm expansion of the PTV for SRS at our institution after 2017.

We were not able to detect differences in RNFS between SRS with concurrent BRAFi/MEKi and other immunotherapies such as pembrolizumab or nivolumab due to the small number of patients and the heterogeneity of systemic therapies. However, an international multicenter retrospective study did report that the incidence of RN was significantly higher in patients treated with SRS and concurrent BRAFi/MEKi than in patients who underwent SRS and other concurrent immunotherapies [31].

Even though the risk of RN after concurrent therapy is similar to that observed with SRS alone, still the possibility of developing RN is higher than treating asymptomatic MBM with TT/IT alone. Therefore, it raises important questions about the optimal treatment strategy and sequencing. As the management paradigm of MBM is rapidly changing, preceding studies had analyzed the use of systemic therapy alone as an initial approach for asymptomatic brain metastases. An open label phase 2 trial reported that ipilimumab showed intracranial activity in some patients with MBM, particularly if metastases were small and asymptomatic. The CNS disease control rate was 10–25% and the CNS objective response rate was 5–16%. The median duration of intracranial PFS of the brain with ipilimumab alone was 1.2–1.9 months [32]. Another phase 2 multicenter randomized phase 2 study investigated the combination of nivolumab and ipilimumab or nivolumab alone in MBM. The combination of nivolumab and ipilimumab showed a better intracranial response than nivolumab alone in patients with asymptomatic MBM (46 and 20%). Median duration of intracranial PFS was not reached in the nivolumab and ipilimumab combination arm and 2.5 months in the nivolumab alone arm [33]. COMBI-MB, an open label multicenter phase 2 trial, analyzed dabrafenib and trametinib in patients with MBM and BRAF^V600^ –mutation. Patients with BRAF^V600E^-positive, asymptomatic melanoma brain metastases, no previous local brain therapy, and good performance status (ECOG 0 or 1) had an intracranial response rate of 58% and a response duration of 6.5 months [8]. These studies showed that TT/IT alone had an effective intracranial activity in patients with MBM. Therefore, it is suggested, that TT/IT alone should be considered as first line therapy in patients with asymptomatic MBM and SRS should be performed in case of progression and symptomatic MBM. This approach might require closer patients’ follow up and more frequent brain MRI. Nevertheless, since TT/IT alone had overall a short duration of intracranial response and as larger tumor volume is a significant predictor for local control after SRS, postponing SRS might compromise the excellent local control rates of SRS [34]. Besides, the risk of RN also increases with the increasing tumor volume and with the consequently rising PTV, V10 and V12 volume [28, 29]. The latest study reported that multiple brain metastases (up to 10 metastases and maximum diameter of 2.5 cm) could be treated in single fraction SRS, which offered an option not just to defer WBRT but also to start TT/IT expeditiously in patients with metastatic melanoma [35, 36].

The limitation of our study is its retrospective characters with small numbers of patients including a heterogeneous patient population. Furthermore, toxicity and follow up data are retrospectively collected from chart review, which limits toxicity analysis. These results warrant prospective evaluation of potential synergistic effects and proper timing between TT/IT with SRS to improve outcomes in patients with MBMs.

## Conclusion

SRS with simultaneous TT/IT was well tolerated. The therapy combination significantly improved LC with no difference in radiation necrosis rate. However, prospective studies on SRS with simultaneous TT/IT are necessary to answer open questions of optimal timing and substances of TT/IT and SRS in combination.

## Data Availability

The datasets used and analysed during the current study are available from the corresponding author on reasonable request.

## Abbreviations

BM: Brain metastasis
CT: Computed tomography
DIC: Distant intracranial control
FET-PET: 18F-Fluoro-Ethyl-Tyrosinepositron-emission tomography
FFF: Flattening-filter-free
GPA: Graded prognostic assessment
KPS: Karnofsky performance score
LC: Local control
LINAC: Linear accelerator
MBM: Melanoma brain metastasis
MRI: Magnetic resonance imaging
OS: Overall survival
RECIST: Response evaluation criteria in solid tumors
RN: Radiation necrosis
RNFS: radiation necrosis-free survival
SRS: Stereotactic radiosurgery
TT/IT: Targeted/ immunotherapy
WBRT: Whole brain radiotherapy
